# Prophylactic cholecystectomy is not mandatory in patients candidate to the resection for small intestine neuroendocrine neoplasms: a propensity score-matched and cost-minimization analysis

**DOI:** 10.1007/s13304-021-01123-2

**Published:** 2021-07-05

**Authors:** Carlo Ingaldi, Laura Alberici, Claudio Ricci, Davide Campana, Cristina Mosconi, Valentina Ambrosini, Giuseppe Lamberti, Lisa Manuzzi, Francesco Minni, Riccardo Casadei

**Affiliations:** 1 Division of Pancreatic Surgery, IRCCS Azienda Ospedaliero-Universitaria Di Bologna, via Albertoni 15, Bologna, Italy; 2 Department of Internal Medicine and Surgery (DIMEC), Alma Mater Studiorum, University of Bologna, Bologna, Italy; 3 Division of Oncology, Azienda Ospedaliero-Universitaria Di Bologna, via Albertoni 15, Bologna, Italy; 4 Division of Radiology, Azienda Ospedaliero-Universitaria Di Bologna, via Albertoni 15, Bologna, Italy; 5 Division of Nuclear Medicine, Azienda Ospedaliero-Universitaria Di Bologna, via Albertoni 15, Bologna, Italy; 6 Department of Specialistic, Diagnostic and Experimental Medicine (DIMES), S.Orsola-Malpighi Hospital, University of Bologna, Bologna, Italy; 7 Center of Excellence for the Diagnosis and Cure of Neuroendocrine Neoplasms Certified By the European Neuroendocrine Tumor Society (ENETS), Berlin, Germany; 8 Pancreas Unit, S.Orsola-Malpighi Hospital, Via Massarenti 9, 40138 Bologna, Italy

**Keywords:** Small intestinal endocrine neoplasm, Cost analysis, Somatostain analogues

## Abstract

To evaluate two competitive strategies in patients undergoing resection of Small-intestine neuroendocrine neoplasms (Si-NEN): prophylactic cholecystectomy (PC) versus On-demand delayed (OC) cholecystectomy. None comparative studies are available. This is a retrospective study based on 247 Si-NENs candidates for the primary tumor resection. Patients were divided into two arms: PC and OC. Propensity score matching was performed, reporting the *d* value. The primary outcome was the rehospitalization rate for any cause. The secondary endpoints were: the rehospitalization rate for biliary stone disease (BSD), the mean number of rehospitalization (any cause and BSD), the complication rate (all and severe). A *P* value < 0.05 was considered significant, and the number needed to treat (NNT) < 10 was considered clinically relevant. Before matching, 52 (21.1%) were in the PC arm and 195 (78.9%) in the OC group. The two arms have a sub-optimal balance for age (*d* = 0.575), symptoms (*d* = 0.661), ENETS TNM stage (*d* = 0.661). After matching, we included 52 patients in PC and 104 in OC one. The two groups are well balanced (all *d* values < 0.5). The rehospitalization rate was similar in the two groups (36% vs 31; *P* = 0.594; NNT = 21). The rehospitalization rate for BSD was lower in the PC arm than OC one (0% vs 7%) without statistical significance (*P* = 0.096) and relevance (NNT = 15). The mean number of readmission (any cause and BSD) and the complication rate (all and severe) were similar. PC was not mandatory in patients having Si-NEN and candidates to the resection of primary tumors.

**Supplementary Information:**

The online version contains supplementary material available at 10.1007/s13304-021-01123-2.

## Introduction

Biliary stone disease (BSD) is common in patients treated with somatostatin analogues (SSA) for Small-intestine neuroendocrine neoplasms (Si-NENs) [1–4]. For this reason, a recent multicentric cohort study suggested the prophylactic cholecystectomy in all patients’ candidates to the resection of the primary tumors. Nevertheless, both European Neuroendocrine Tumors Society (ENETS) [5] and North American Endocrine Tumors Society (NANETS) [6] guidelines do not strongly recommend a prophylactic cholecystectomy during primary tumor resection. Uncertainty and contradictory positions are due to the absence of high quality and comparative studies. In the past 25 years, only a non-comparative study was published about this topic. Indeed, Norlen et al. [7] reported that the incidence of gallstone-related complications was higher than in the general population, and for this reason, they recommend prophylactic cholecystectomy. However, the authors did not perform any comparison between on-demand delayed versus prophylactic cholecystectomy.

Thus, our study aimed to fill this gap by comparing two different strategies: on-demand delayed cholecystectomy (OC) for symptomatic biliary stone disease versus prophylactic cholecystectomy (PC) during the primary tumor resection. A propensity score matching (PSM) analysis was planned to minimize the selection bias. The primary endpoint was the rate of rehospitalization after primary tumor resection in the two groups. The secondary endpoints were the following: the rate of rehospitalization for BSD, the mean number of rehospitalization for any cause and BSD, and the complication rate and severity, the mean difference in rehospitalization due to BSD and any cause. We also evaluated the economic consequences of the two different strategies performing a cost-minimization analysis (CMA).

## Methods

### Study design

A retrospective study was carried out using a prospectively maintained database of patients treated at our Center of Excellence to diagnose and cure NENs certified by the European Neuroendocrine Tumor Society (ENETS). The database included all patients observed from January 2000 to December 2019 with a diagnosis of Si-NENs. All patients at the first visit or follow-up signed a non-specific written consent for retrospective non-interventional studies. Data collection, analysis and results were obtained using the principles of the STROCSS guidelines [8] The study’s inclusion criteria were the following: (1) patients with a diagnosis of Si-NEN; (2) resection of the primary tumor with or without concomitant cholecystectomy; (3) absence of a history of a biliary stone disease or cholecystectomy before Si-NEN diagnosis. The following data were extracted for the matching: sex, age, comorbidity, presence of symptoms, type of surgery (emergency or elective), ENETS TNM stage [9], WHO 2019 grading [10] of the primary tumor, type of resection (R0/1 vs R2), administration of SSA therapy, duration of follow-up. The following parameters were compared: (a) the rate and the mean number of rehospitalization after primary tumor resection, defined as any new hospital admission for any cause; (b) the rate and the mean number of rehospitalization for the BSD, including biliary colic, cholecystitis, biliary pancreatitis, choledocholithiasis, and cholangitis; (c) the complication rate and severity after surgery classified by Clavien–Dindo score (CD) [11]; (d) overall and specific costs (the costs related to the surgery of Si-NENs tumors, and the costs for all types and BSD of rehospitalizations). The analysis was conducted using the intention to treat principle, including in the OC arm, all patients with and without BSD or need of cholecystectomy. For the OC arm, the complication rate included the sum of events that occurred in both procedures.

### Statistical analysis

All parameters were reported as frequencies and percentage, median and interquartile range (IQR) or mean and standard deviation (SD). The PSM was performed using logistic regression. The two groups were matched for the following parameters: sex, age, comorbidity, presence of symptoms, type of surgery, ENETS TNM stage, WHO 2019 grading of the primary tumor, type of resection, SSA-therapy, and duration of follow-up. The PSM method is closest to the neighborhood method having a caliper width of 0.20 pooled standard, and the matching was created with a 2:1 ratio. Standardized mean difference (*d* value) was used to assess the balance between the two groups. A *d* value < 0.2 indicates a small difference between the two groups, identifying an excellent balance. A *d* value between 0.2 and 0.5 shows a medium difference, implying a good balance. A *d* value between 0.5 and 0.8 shows a high difference, implying a sub-optimal balance. A *d* value > 0.8 resulted in a remarkable difference, suggesting a poor balance between the two groups. [12] The outcomes were also reported describing the number needed to treat (NNT) in adopting PC strategy instead of OC. The NNT was reported with a confidence interval of 95%. A negative value of NNT or within 95% CI indicates that the PC results in harmful [13]. When the NNT with 95% CI assumes positive value was interpreted as follows. Considering that the cholecystectomy was not a life-saving procedure, we considered that PC was very useful if NNT ≤ 1 [14]. The PC was considered moderately effective for NNT values between 1 and 10 and clinical useless for NNT > 10. The CMA analysis was conducted following the EVEREST guidelines [15]. The costs were assessed from the healthcare providers’ perspective and were extracted from the current payments within the Italian National Health, converted to the 2020-euro equivalent. [16]. A Monte Carlo probabilistic analysis was used to investigate the strategies’ economic impact, simulating different scenarios. In each scenario, we hypothesized different rehospitalization rates from 0 to 100%. The burden of biliary disease in total rehospitalization was maintained the same as our cohort. The number of distribution samples of the Monte Carlo simulation was set at 3000 for each strategy arm, simulating a randomized clinical trial with 6000 patients for each cost scenario. The cost-analysis diagram model was plotted as follows: the blue square was the decision node; the green circles were the chance node; the red triangles were the terminal nodes. The cost distribution was obtained from our cohort for PC arm, no rehospitalization; PC arm, rehospitalization; OC arm, no rehospitalization; OC arm, rehospitalization. Statistical analysis was carried out using Fisher’s exact test, Student’s *T* test, and Pearson Chi-square test. Two-tailed *P* values inferior to 0.05 were considered statistically significant. Standardized mean difference (*d* value) was used to assess the two groups’ economic differences in the Montecarlo simulation. All statistical analyses were run with Statistical Package for Social Science (SPSS) version 21, STATA 14, and TreeAge Pro 2011.

## Results

### Patients included

**Fig. 1:**
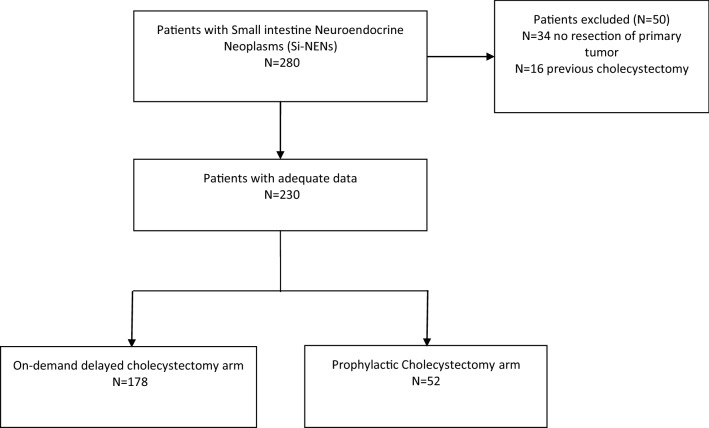
Flow-diagram of the selection process

Figure 1 shows the selection process. A total of 280 records were screened, but only 230 patients, who meet all inclusion criteria, were considered in the final analysis: 178 (77.4%) in the OC and 52 (22.6%) in the PC group. During the median follow-up of 61 (27–124) months, only 29 patients (16.3%) of the OC group developed lithiasis, and amongst these, only 16 patients (8.9%) underwent cholecystectomy due to symptomatic BSD. On the contrary, no choledocholithiasis or other biliary diseases were observed in the PC group. It should be noted that none of the cholecystectomies was performed in an emergency setting in the OC group.

### Unmatched population

Clinical and demographic variables are reported in Supplementary Table 1. No significant or large differences were found between the two groups regarding sex, age, comorbidity, type of surgery, 2019 WHO grading, type of resection, or mean follow-up after primary tumor resection. Symptoms tumor-related were more frequently in OC than PC group (69.6% vs. 53.8%, *P* = 0.045). Comparing the two arm for the ENETS TNM stage distribution, we observed several patients in stage IV in the PC group (75% vs 42.7%; *P* < 0.001). Both groups showed a high percentage of patients treated with SSA therapy, with a statistically significant difference favoring the PC group (76.9% vs 60.0%, *P* = 0.033). Some of these differences suggesting a sub-optimal (symptoms with *d* = 0.373 and SSA therapy with *d* = 0.424) or poor balance (ENETS TNM stage; *d* = 0.694). The outcomes are reported in Supplementary Table 2. The rehospitalization rate for any cause was similar in the two groups (41.6% vs 30.8%; *P* = 0.197). Indeed, PC strategy was related in an NNT = 9, but it could result harmful in the worst scenario (− 23 to 3). The re-hospitalization rate for BSD was lower PC arm (8.9% vs. 0%; *P* = 0.026). The PC strategy had an NNT = 11 (6–278), suggesting a useless effect in preventing BSD rehospitalization. The mean number of new rehospitalization was 1.1 ± 1.8 and 0.6 ± 1.2 (*P* = 0.083) in the OC and PC groups, respectively. The mean number of hospitalization for the BSD was similar in both groups, 0.2 ± 0.6 vs 0 (*P* = 0.061) in OC and PC groups, respectively. The rate and the severity of complications after primary tumor resection were similar with or without prophylactic cholecystectomy. No relevant clinical benefit was observed for PC strategy in surgical-related for all (NNT = 16; − 14 to 5, 95% CI) and severe complications (NNT = 67; − 25 to 14, 95% CI). The total costs were similar for the two groups (18,580 vs 19,684 euro for OC and PC, respectively; *P* = 0.282). The primary tumor resection costs were higher in the PC than in OC (17,842 vs 14,758 Euro; *P* < 0.001). The costs of rehospitalization for all-cause showed a tendency without statistical significance in favor of PC (1841 vs 3822 Euro; *P* = 0.054, in PC and OC, respectively). The rehospitalization costs for BSD were lower in PC (0 vs 370 Euro) without statistical significance (*P* = 0.071).

### Matched population

The demographic and clinical characteristics of the matched population are shown in Table 1. A total of 156 patients were included for the analysis, 104 for the OC group and 52 for the PC group. No sub-optimal or poor balancing was observed after PSM. Outcomes are reported in Table 2.

**Table 1:** Demographic, clinical characteristics of the matched population of patient resected for Si-NEN

Factors	*N* (%) or Median (IQR)			
	OC (104)	PC (52)	*P* value	*d* value
Sex			0.387	0.177
Male	66 (63.5)	29 (55.8)		
Female	38 (36.5)	23 (44.2)		
Age (years)	60 (50–68)	65 (55–70)	0.100	0.031
Comorbidity			0.571	0.131
No	27 (26)	16 (30.8)		
One or more	77 (74)	36 (69.2)		
Symptoms			0.161	0.289
No	35 (33.7)	24 (46.1)		
Yes	69 (66.3)	28 (53.9)		
Type of surgery			0.519	0.214
Elective	82 (78.9)	44 (84.6)		
Emergency	22 (21.1)	8 (15.4)		
ENETS TNM stage			0.930	0.039
I	0	0		
II	2 (1.9)	1 (1.9)		
III	26 (25.0)	12 (23.1)		
IV	76 (73.1)	39 (75.0)		
SSA therapy			0.350	0.216
No	32 (30.8)	12 (23.1)		
Yes	72 (69.2)	40 (76.9)		
2019 WHO grading			0.255	0.278
G1	72 (69.2)	41 (78.8)		
G2	32 (30.8)	11 (21.2)		
Type of resection			0.396	0.196
R0/1	49 (47.1)	29 (55.8)		
R2	55 (52.9)	23 (44.2)		
Follow-up (months)	54 (26–124)	71 (33–122)	0.873	0.028

*N* number, *IQR* interquartile range, *OC* on-demand delayed cholecystectomy, *PC* upfront cholecystectomy, *ENETS* European Neuro-Endocrine Tumors Society, *TNM* tumor nodes metastasis, *SSA* somatostatin analogues, *WHO* World Health Organization, *R0* radical resection with no microscopic residual of disease, *R1* radical resection with a microscopical residual of disease, *R2* resection with a macroscopical residual of disease, *Si-NEN* small intestine neuroendocrine neoplasm

**Table 2:** Post-operative characteristics of the matched population of patient resected for Si-NEN

Outcomes	*N* (%) or mean (SD)			
	OC (104)	PC (52)	*P* value	NNT (PC vs. OC)
Hospital re-hospitalization for any cause			0.593	18 (− 10^a^ to 5)
No	66 (63.5)	36 (69.2)		
Yes	38 (36.5)	16 (30.8)		
Hospital re-hospitalization for BSD			0.096	15 (− 100^a^ to 8)
No	97 (93.3)	52 (100)		
Yes	7 (6.7)	0 (0)		
Number of re-hospitalization for any cause (mean; SD)	1.1 (1.9)	0.6 (1.2)	0.158	-
Number of re-hospitalization for BSD (mean; SD)	0.1 (0.5)	0	0.104	-
Complications (C–D)			0.297	21 (− 5^a^ to 10)*
No	79 (75.9)	37 (71.1)		
1	7 (6.7)	1 (1.9)		
2	14 (13.5)	13 (25.0)		
3	3 (2.9)	1 (1.9)		
4	1 (0.9)	0		
Severe complications (C–D ≥ 3)			0.665	50 (− 25^a^ to 13)
No	100 (96.1)	51 (98.1)		
Yes	4 (3.9)	1 (1.9)		
Total cost (mean; SD; Euro)	18,434 (7419)	19,684 (4401)	0.264	–
Cost due to primary surgery (mean; SD; Euro)	14,758 (0)	17,842 (380)	< 0.001	–
Total cost for all type of re-hospitalization (mean; SD; Euro)	3676 (7419)	1841 (4357)	0.101	–
Total cost for BSD re-hospitalization (mean; SD; Euro)	275 (1229)	0	0.108	–

*N* number, *SD* Standard deviation, *OC* on-demand delayed cholecystectomy, *PC* prophylactic cholecystectomy, *NNT* number needed to treat, *BSD* Biliary stone disease, *C–D* Clavien–Dindo classification

*Not computable

^a^The strategy resulted in a harm

Regarding the primary endpoint, the rate of patients who experienced a new hospital admission were 36.5% vs 30.8% (difference + 5.7%, *P* = 0.514) in OC and PC groups, respectively. The PC strategy did not provide a clinically relevant advantage (NNT = 18; − 10 to 5, 95% CI). The rehospitalization rate for BSD was 6.7% for OC patients and 0 for PC ones (*P* = 0.096), considering only the biliary disease (NNT = 15; − 100 to 8, 95% CI). The mean number of rehospitalization for any cause was similar (1.0 ± 1.9 vs 0.6 ± 1.2; *P* = 0.158) in OC and PC groups, respectively. The mean number of rehospitalization for BSD was close to 0 in both groups: 0.1 ± 0.5 vs 0; *P* = 0.104, in OC and PC groups, respectively. The rate of patients with uneventful postoperative stay was 75.1% vs 71.1% (*P* = 0.297) in OC and PC groups. The rate of severe complications (grade ≥ 3 by CD) was 3.9% in the OC group and 1.9% in PC one (*P* = 0.665). No relevant clinical benefit was observed for PC strategy for all (NNT = 21; − 5 to 10, 95% CI) and severe complication (NNT = 50; − 25 to 13, 95% CI). The total costs were similar for the two groups (18,434 vs 19,684 euro for OC and PC, respectively; *P* = 0.264). The primary tumor resection costs were higher in the PC than in OC (17,842 vs 14,758 Euro; *P* < 0.001). The costs of rehospitalization for all-cause showed a tendency without statistical significance in favor of PC (1841 vs 3676 Euro; *P* = 0.101, in PC and OC, respectively). The rehospitalization costs for BSD were lower in PC (0 vs 275 Euro) without statistical significance (*P* = 0.108).

### CMA analysis

**Fig. 2:**
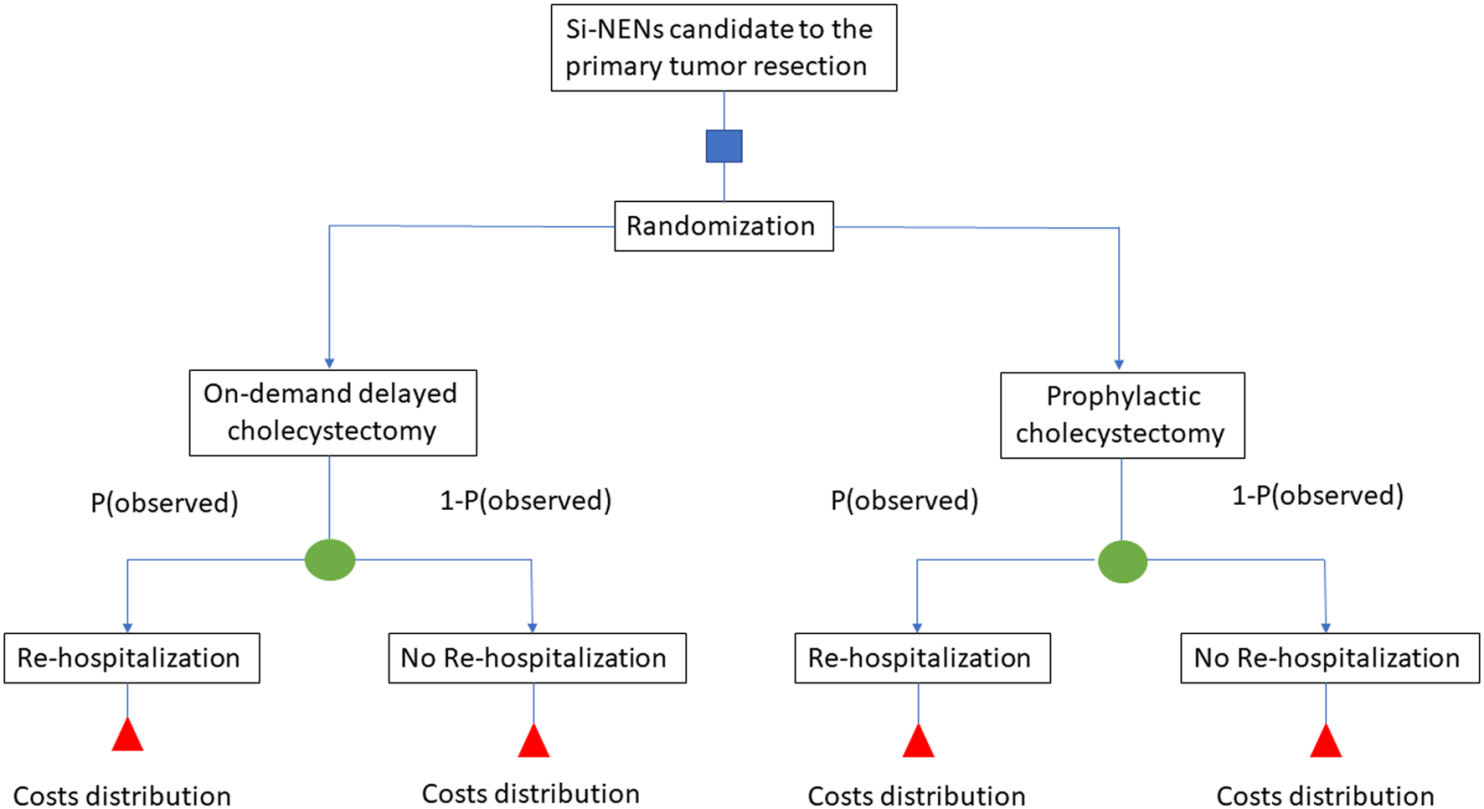
Schematic representation of the model used in the CMA analysis. Blue square represents the decision node. The number of patients simulated beyond the blue square was 3000 for each arm. The green circle represents the chance node. P, the probabilities were represented for patients who experienced the rehospitalization and 1-P for patients who did not experience the rehospitalization. For the base case scenario, the observed probabilities were used (36.5% for OC arm and 30.8% for PC arm). The red triangle represents the terminal node. The costs were calculated using the Italian National Health’s current payments, converted to the 2020-euro equivalent [15]. We hypothesized that the costs have triangular or uniform distribution and were the following: PC arm, no rehospitalization, uniform distribution = min. 17,790–max. 20,533 Euro; PC arm, re-hospitalization, triangular distribution = min. 18,718, median = 23,947 Euro, max. 41,578 Euro; OC arm, no re-hospitalization, uniform distribution = min. 14,758–max. 15,793 Euro; OC arm, re-hospitalization, triangular distribution = min. 15,686, median = 23,951 Euro, max. 50,195 Euro (Color figure online)

**Fig. 3:**
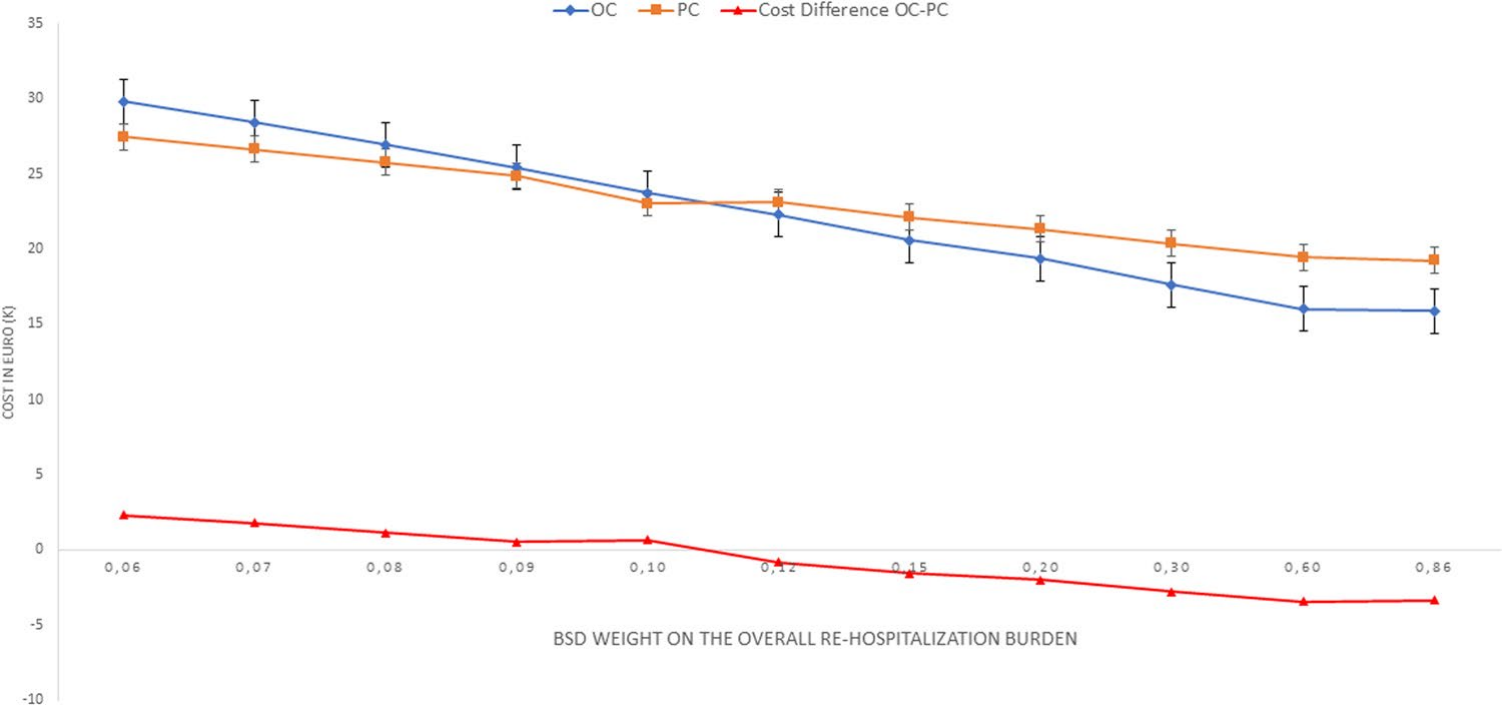
Schematic representation of the one-way sensitivity analysis for CMA multiple scenarios. *Y*-axis represents the costs in Euro; *X*-axis represents the percentage of rehospitalization for biliary disease

The CMA analysis was reported in Table 3 and plotted in Figs. 2, 3. The Montecarlo simulation suggested that OC was the most expensive strategy in a setting with a high risk of rehospitalization (> 50%). The difference was large in very high-risk setting (99% to 90%, + 2321 Euro, *d* value = 1.265). The difference was medium (*d* value = 0.211) for overall hospitalization rate equal to 80% and small for value < 80%. On the contrary, the OC approach’s costs were lower when the risk of rehospitalization was inferior to 50%. In a scenario with a rehospitalization rate similar to our cohort (40%), the OC approach permits us to gain near 1542 Euro for each patient managed with a medium difference (*d* value = 0.351). For rehospitalization rate inferior to 10% (low-risk setting), the OC was the less expensive strategy with a remarkable difference with PC in terms of overall costs (> 3429 Euro; *d* value > 0.800). Figure 3 showed that the OC strategy was less expensive than the PC one, only when the BSD weighed the overall rehospitalization burden. In other words, in a setting where the rehospitalization rate was high, a PC strategy did not minimize the costs.

**Table 3:** CMA analysis using 3000 Montecarlo simulation per arm

Overall Hospitalization rate, scenarios for OC arm (%)*	OC costs, mean (SD), Euro	PC costs, mean (SD), Euro	Δ OC-PC in Euro	*d* value
100	29,792 (151)	27,471 (225)	2321	1.265
90	28,425 (4555)	26,650 (3272)	1775	0.566
80	26,916 (6066)	24,861 (4283)	1155	0.211
70	25,393 (6957)	24,861 (4283)	532	0.196
60	23,707 (747)	23,032 (424)	675	0
50	22,285 (7592)	23,089 (4427)	− 804	0.175
40^a^	20,574 (7382)	22,116 (4198)	− 1542	0.351
30	19,349 (6974)	21,337 (3830)	− 1988	0.422
20	17,592 (5917)	20,353 (3035)	− 2761	0.727
10	16,003 (4166)	19,432 (1529)	− 3429	1.029
5.7	15,881 (3975)	19,238 (826)	− 3357	1.886

Δ = Difference in costs between OC and PC arm; *d* value: small mean difference. A *d* value < 0.2 indicates a minimal difference between the two groups. A *d* value between 0.2 and 0.5 shows a small difference. A *d* value between 0.5 and 0.8 shows a medium difference. A *d* value > 0.8 resulted in a remarkable difference

*OC* on-demand delayed cholecystectomy, *SD* standard deviation, *PC* prophylactic cholecystectomy

*For the PC arm, we assumed in each scenario a − 5.7% rehospitalization rate, as reported in Table 2

^a^Rate of rehospitalization near to the clinical scenario observed in our cohort

## Discussion

The present study suggested that, in patients having Si-NENs, the PC is not mandatory with the primary tumor resection. Indeed, the OC had similar clinical results to the PC strategy with some economic advantages. This evidence is sustained by a comparative study in a large retrospective ENETS cohort for the first time. Indeed, we retrospectively analyzed our experience, dividing the patients into two groups representing the two competitive strategies: prophylactic cholecystectomy in “all patients” versus an “on-demand delayed cholecystectomy” only in patients with symptomatic BSD. It should be noted that the OC strategy was the same routinely used in the general population in which the cholecystectomy was performed when the BSD-related symptoms appeared [17]. Some methodological and statistical precautions have been taken to overcome the selection bias due to retrospective design. Firstly, the intention to treat principle was adopted, including in the OC arm, all patients with and without cholecystectomy during the follow-up. Secondly, we used the PSM analysis to minimize the difference between the two groups due to retrospective design and bias selection. Thirdly, the results were described using the NNT to report the clinical impact of the two strategies. Fourthly, the cost-minimization analysis was planned to consider the economic aspects.

The first interesting observation was that the overall rehospitalization rate was similar in both arms (36% vs 31%, OC, and PC, respectively). Thus, the PC strategy’s clinical advantages are marginal, avoiding only one rehospitalization every 18 cholecystectomies performed. Moreover, this advantage disappeared in the worst scenario because the NNT assumes negative values within the 95% CI. Considering only the readmission for BSD, we observed a difference in favor of the PC arm (7% vs 0%) with a statistical trend. Nevertheless, the PC strategy’s impact remained clinically not relevant, avoiding only one readmission every 15 cholecystectomies performed. Finally, the complication rate and the severity related to the two strategies were very similar. These results suggested that BSD’s weight in the mean rehospitalization rate of Si-NENs patients was very low. In other words, in most of the patient candidate for Si-NENs resection, a PC was useless because the BSD disease will represent a marginal reason for the rehospitalization. Thus, the OC and PC strategies were similarly efficacious and safe. This datum did not surprise because, as previously reported by Brighi et al. [1], the BSD was mainly related to SSA therapy. In our series, nearly one-third of patients did not receive SSA therapy because it was unnecessary during the follow-up. These patients can be considered “not more at risk” to the general population for BSD at the time of surgery. Moreover, Brighi et al. [1] suggested that only 27% of patients receiving SSA developed a BSD, further reducing the quote of patients “at-risk” for BSD at the time of surgery.

In other words, the PC strategy could be useful only for a minority of the patients. A CMA evaluation seems suitable in this setting because the two strategies are very similar in efficacy and safety. The CMA should be useful in establishing the treatment less expensive for the health care system. We compared the total cost of OC and PC strategy, observing that the PC arm’s total costs were higher than OC ones (19,684 vs 18,434 Euro; *P* = 0.264), but this difference was not statistically relevant. The reason for them was that the PC costs related to the surgery were higher than OC (14,758 vs 17,842 Euro; *P* < 0.001). The Montecarlo simulation confirmed that, in a large population, the PC advantages are limited, and, in intermediate scenarios, this strategy could be more expensive than the OC one.

From an economic perspective, the PC strategy was useful only in patients with a high rehospitalization rate. In other words, if our patient had a high risk for rehospitalization (e.g., metastatic disease or palliative resection), then the PC could produce some advantages reducing the costs for the health care system. On the contrary, in patients with a low risk of rehospitalization ( e.g., localized neoplasm or radical resection), the BSD’s economic weight, per se, was low in the overall costs, and PC strategy seems to be too expensive for the Health care system.

The current study has some significant limitations: the retrospective and single-center design and the long observation period, only partially mitigated by intention to treat analysis, PSM approach, and use of NNT. Another limitation was that the primary endpoint is time-depending. The median observation time was 61 months, and the externalization of the results could be partially limited. Nonetheless, the “OC strategy” did not impose never performing the cholecystectomy but operating the patients only when symptomatic, similarly to the general population. Finally, several surgeons were involved in the procedures. However, all surgeons have completed the learning curve for the cholecystectomy, and all procedures were performed in high-volume hepato-biliary-pancreatic referral center.

In conclusion, the present study compared, for the first time, two competitive strategies in patients affected by Si-NEN. The on-demand surgery can be considered non-inferior to the prophylactic cholecystectomy in these patients, even in those candidates to receive SSA. This approach could avoid several useless and expensive cholecystectomies. All these results should be confirmed in prospective, large, and multicentric studies.

Provenance and peer review not commissioned, externally peer-reviewed.

## Supplementary Information

Below is the link to the electronic supplementary material.

Supplementary file1 (DOCX 18 KB)

Supplementary file2 (DOCX 18 KB)
