# Supplementary material for: Prophylactic cholecystectomy is not mandatory in patients candidate to the resection for small intestine neuroendocrine neoplasms: a propensity score-matched and cost-minimization analysis
Source: Updates Surg. 2021 Jul 5;74(3):991–8. doi: 10.1007/s13304-021-01123-2 (PMC9213268; doi:10.1007/s13304-021-01123-2)
Supplement: Supplementary file 1 — Supplementary file1 (DOCX 18 KB) [file 13304_2021_1123_MOESM1_ESM.docx]

**Table 1. Demographic, clinical characteristics of the unmatched population of patient resected for Si-NEN**

| **Factors** | **N(%) or Median (IQR)** | |  | | |
| --- | --- | --- | --- | --- | --- |
|  | **OC (178)** | **PC (52)** | **P-value** | | **d-value** |
| **Sex**  M  F | 109 (61.2)  68 (38.8) | 29 (55.8)  23 (44.2) | 0.521 | 0.132 | |
| **Age (years)** | 62 (54 to 70) | 65 (55 to 70) | 0.581 | 0.001 | |
| **Comorbidity**  No  One or more | 48 (26.9)  130 (73.1) | 16 (30.8)  36 (69.2) | 0.860 | 0.051 | |
| **Symptoms**  No  Yes | 54 (30.3)  124 (69.6) | 24 (46.2)  28 (53.8) | 0.045 | 0.373 | |
| **Type of surgery**  Elective  Emergency | 133 (74.7)  45 (25.3) | 44 (84.6)  8 (15.4) | 0.189 | 0.342 | |
| **ENETS TNM Stage**  I  II  III  IV | 2 (1.1)  15 (8.4)  85 (47.8)  76 (42.7) | 0  1 (1.9)  12 (23.1)  39 (75) | <0.001 | 0.694 | |
| **SSA therapy**  No  Yes | 70 (39.3)  108 (60.7) | 12 (23.1)  40 (76.9) | 0.033 | 0.424 | |
| **2019 WHO Grading**  G1  G2 | 121 (67.9)  57 (32.1) | 41 (78.9)  11 (21.1) | 0.250 | 0.311 | |
| **Type of resection**  R0/1  R2 | 117 (65.7)  61 (34.3) | 29 (55.8)  23 (44.2) | 0.195 | 0.269 | |
| **Follow-up (months)** | 67 (31 to 122) | 71 (33 to 122) | 0.861 | 0.001 | |

**Legend:** N= number; IQR= Interquartile range; OC= on-demand delayed cholecystectomy; PC=upfront cholecystectomy; M= male; F= female; ENETS= European Neuro-Endocrine Tumors Society; TNM= Tumor Nodes Metastasis; SSA= Somatostatin analogues; WHO= World Health Organization; R0= radical resection with no microscopic residual of disease; R1= radical resection with a microscopical residual of disease; R2= resection with a macroscopical residual of disease; Si-NEN= Small intestine neuroendocrine neoplasm.
