# Supplementary material for: Prophylactic cholecystectomy is not mandatory in patients candidate to the resection for small intestine neuroendocrine neoplasms: a propensity score-matched and cost-minimization analysis
Source: Updates Surg. 2021 Jul 5;74(3):991–8. doi: 10.1007/s13304-021-01123-2 (PMC9213268; doi:10.1007/s13304-021-01123-2)
Supplement: Supplementary file 2 — Supplementary file2 (DOCX 18 KB) [file 13304_2021_1123_MOESM2_ESM.docx]

**Table 2. Post-operative characteristics of the unmatched population of patient resected for Si-NEN**

| **Outcomes** | **N(%) or mean (SD)** | |  | |
| --- | --- | --- | --- | --- |
|  | **OC (178)** | **PC (52)** | **P-value** | **NNT (PC vs. OC)** |
| **Hospital re-hospitalization for any cause**  No  Yes | 104 (58.4)  74 (41.6) | 36 (69.2)  16 (30.8) | 0.197 | 9 (-23 to 3) |
| **Hospital re-hospitalization for BSD**  No  Yes | 162 (91.1)  16 (8.9) | 52 (100)  0 (0) | 0.026 | 11 (6 to 278) |
| **Number of re-hospitalization for any cause (mean; SD)** | 1.1 (1.8) | 0.6 (1.2) | 0.083 | - |
| **Number of re-hospitalization for BSD (mean; SD)** | 0.2 (0.6) | 0 | 0.060 | - |
| **Complications (C-D)**  No  1  2  3  4 | 138 (77.5)  12 (6.7)  22 (12.4)  5 (2.8)  1 (0.6) | 37 (71.1)  1 (1.9)  13 (25.0)  1 (1.9)  0 | 0.164 | 16 (-14 to 5)* |
| **Severe Complications (C-D ≥3)**  No  Yes | 172 (96.6)  6 (3.4) | 51 (98.1)  1 (1.9) | 1.000 | 67 (-25 to 14) |
| **Total cost (mean; SD; Euro)** | 18580 (6987) | 19684 (4401) | 0.282 | - |
| **Cost due to primary surgery (mean; SD; Euro)** | 14758 (0) | 17842(380) | <0.001 | - |
| **Total cost for all types of re-hospitalization (mean; SD; Euro)** | 3822 (6987) | 1841 (4357) | 0.054 | - |
| **Total cost for BSD re-hospitalization (mean; SD; Euro)** | 370 (1468) | 0 | 0.071 | - |

**Legend: N=** number; **SD=** Standard deviation; **OC**= on-demand delayed cholecystectomy; **PC=** prophylactic cholecystectomy; **NNT=** number needed to treat; **NNH=** number needed to harm; **BSD=** Biliary stone disease; **C-D=** Clavien–Dindo classification; * calculated for complication (No vs. Yes); ^= the strategy resulted in a harm
